# L-Carnitine Is an Endogenous HDAC Inhibitor Selectively Inhibiting Cancer Cell Growth *In Vivo* and *In Vitro*

**DOI:** 10.1371/journal.pone.0049062

**Published:** 2012-11-05

**Authors:** Hongbiao Huang, Ningning Liu, Haiping Guo, Siyan Liao, Xiaofen Li, Changshan Yang, Shouting Liu, Wenbin Song, Chunjiao Liu, Lixia Guan, Bing Li, Li Xu, Change Zhang, Xuejun Wang, Q. Ping Dou, Jinbao Liu

**Affiliations:** 1 Protein Modification and Degradation Lab, Department of Pathophysiology, Guangzhou Medical College, Guangdong, People's Republic of China; 2 Experimental Medical Research Center, Guangzhou Medical College, Guangzhou, Guangdong, People's Republic of China; 3 Department of Hematology, The People's Hospital of Guangxi Autonomous Region, Nanning, Guangxi, People's Republic of China; 4 Division of Basic Biomedical Sciences, Sanford School of Medicine of the University of South Dakota, Vermillion, South Dakota, United States of America; 5 The Molecular Therapeutics Program, Barbara Ann Karmanos Cancer Institute, and Departments of Oncology, Pharmacology and Pathology, School of Medicine, Wayne State University, Detroit, Michigan, United States of America; Peking University Health Science Center, China

## Abstract

L-carnitine (LC) is generally believed to transport long-chain acyl groups from fatty acids into the mitochondrial matrix for ATP generation *via* the citric acid cycle. Based on Warburg's theory that most cancer cells mainly depend on glycolysis for ATP generation, we hypothesize that, LC treatment would lead to disturbance of cellular metabolism and cytotoxicity in cancer cells. In this study, Human hepatoma HepG2, SMMC-7721 cell lines, primary cultured thymocytes and mice bearing HepG2 tumor were used. ATP content was detected by HPLC assay. Cell cycle, cell death and cell viability were assayed by flow cytometry and MTS respectively. Gene, mRNA expression and protein level were detected by gene microarray, Real-time PCR and Western blot respectively. HDAC activities and histone acetylation were detected both in test tube and in cultured cells. A molecular docking study was carried out with CDOCKER protocol of Discovery Studio 2.0 to predict the molecular interaction between L-carnitine and HDAC. Here we found that (1) LC treatment selectively inhibited cancer cell growth *in vivo* and *in vitro*; (2) LC treatment selectively induces the expression of p21^cip1^ gene, mRNA and protein in cancer cells but not p27^kip1^; (4) LC increases histone acetylation and induces accumulation of acetylated histones both in normal thymocytes and cancer cells; (5) LC directly inhibits HDAC I/II activities *via* binding to the active sites of HDAC and induces histone acetylation and lysine-acetylation accumulation *in vitro*; (6) LC treatment induces accumulation of acetylated histones in chromatin associated with the p21^cip1^ gene but not p27^kip1^ detected by ChIP assay. These data support that LC, besides transporting acyl group, works as an endogenous HDAC inhibitor in the cell, which would be of physiological and pathological importance.

## Introduction

Carnitine is biosynthesized from the amino acids lysine and methionine and its biologically active form is L-carnitine (LC). It is generally believed that carnitine transports long-chain acyl groups from fatty acids into the mitochondrial matrix, where they can be broken down through β-oxidation to acetyl-CoA to obtain usable energy *via* the citric acid cycle [1]–[3]. Therefore LC is required for the generation of metabolic energy in living cells.

It has been well known that most cancer cells predominantly generate energy by a high rate of glycolysis followed by lactic acid fermentation in the cytosol, rather than by a comparatively low rate of glycolysis followed by oxidation of pyruvate in mitochondria like most normal cells. This is known as Warburg's effect in cancer cells [4], [5]. Rapidly growing malignant cells typically have glycolytic rates that are up to 200 times higher than those of their normal tissues of origin. Even though Warburg effect has been challenged and further developed, this theory remains the most frequently cited evidence that tumors display dysfunctional metabolism [6].

Based on this theory that the citric cycle is detrimental in most cancer cells [7], [8], we hypothesize that LC would lead to disturbance of cellular metabolism in cancer cells but not in normal cells. In this study, we investigated the effects of LC on cytotoxicity both in cancer and normal cells. We found that LC selectively inhibited cancer cell growth both *in vitro* and *in vivo*. We further investigated the mechanism of LC-mediated cytotoxicity and found that physiological concentrations of LC could directly inhibit HDAC activities.

## Materials and Methods

### Materials and agents

LC, R-carnitine, oligomycin (No. 04876), butyrate (Buty) and trichostatin A (TSA) were purchased from Sigma-Aldrich (St. Louis, MO). HDAC^TM^ I/II assay and screening system was purchased from Promega corporation (Madison, WI). L-glucose and D-glucose were obtained from Alfa Aesar (Karlsruhe, Germany). Fetal bovine serum (FBS) was purchased from Invitrogen Co. (Carlsbad, CA). Rabbit monoclonal antibodies against Acetyl-H3 (Lys9) (C5B11), rabbit polyclonal antibodies against PARP, acetylated-Lysine, acetyl-H4 (Lys8), and mouse monoclonal antibodies against p21 ^Waf1/Cip1^ (DCS60) were all purchased from Cell Signaling (Beverly, MA). Antibodies against Rb (G401), Phospho-Rb (S780) were purchased from Bioworld Technology, Inc. Mouse monoclonal antibody p27 (F-8), rabbit polyclonal antibodies against GAPDH (FL-335) and horseradish peroxidase (HRP)-labeled secondary antibodies were from Santa Cruz Biotechnology Inc. (Santa Cruz, CA). Enhanced chemiluminescence reagents were purchased from Amersham Biosciences (Piscataway, NJ).

### Mice and diet

Male normal Balb/c and Balb/c nude mice aged 5 weeks (18–22 g) were purchased respectively from Guangdong Animal Center and housed in stainless steel wire-mesh cages in an animal room at constant temperature with a 12-h-light/-dark cycle. The mice consumed a commercial nonpurified diet and water ad libitum. All experimental protocols were in accordance with the Guangdong Animal Center for the ethical treatment of animals and approved by the Animal experimental Committee of Guangzhou Medical College (SCXK2008-2002). Male normal Balb/c mice were randomly assigned to two groups (n = 8 mice/group) and were *i.p*. injected with either vehicle (saline) or LC at 400 mg/kg for consecutive 15 days. Body weight and organ weight were detected and summarized. Male Balb/c nude mice were *s.c*. inoculated in the left armpit with HepG2 cells (1×10^6^ cells/mouse). When the tumor size reaches 50–75 mm^3^, mice were randomly divided into two groups (n = 8 mice/group) and were *i.p* injected with either vehicle (saline) or LC (400 mg/kg, once/day) for 15 days except day 8. Body weight and tumor weight were detected and summarized. To better illustrate these results, all the primary data were changed to the relative level (%).

### Cell culture

Human hepatoma HepG2 and SMMC-7721 cell lines, purchased from American Type Culture Collection (Manassas, VA), were grown in RPMI 1640 supplemented with 10% FBS, 100 U/mL benzyl penicillin and 100 U/mL of streptomycin, pH 7.4 in a humidified atmosphere with 5% CO_2_ at 37°C. Thymocyte isolation and primary culture was performed as follows: briefly, the thymus from Balb/c mouse was rolled on a piece of sterile gauze to remove attached fat and connective tissue and the gland was placed into a 60 mm Petri dish containing 5 mL of cold media (HBSS at pH 7.0 containing 5% fetal calf serum at 4°C) and gently teased the thymus apart with needles and pipeted up and down several times to carefully break up any cell clumps. The mixtures were passed through a 75 μm stainless mesh to remove clumps of tissue, centrifuged the cell suspensions (250 g, 10 min, 4°C) and resuspended the cell pellets with 5 mL RPMI 1640 medium. The total cell numbers were counted with trypan blue in a white blood cell counter.

### Cell death assay

This was performed using Annexin V-FITC and propidium iodide (PI) double staining, followed by flow cytometry as previously described [9]. In brief, cultured HepG2 cells were harvested and washed with cold PBS and resuspended with the binding buffer, followed by Annexin V-FITC incubation for 15 min and PI staining for another 15 min at 4°C in dark. The stained cells were analyzed with flow cytometry within 30 min.

### ATP content determination

ATP content determination was performed as described previously [10]. Briefly, Equal number of cultured cells was collected and the cell pellet was immediately frozen and stored in liquid nitrogen for subsequent ATP analyses. The lysates were centrifuged at 12 000 r.p.m. for 10 min at 4°C. The supernatant was collected for analyzing ATP by using a reversed-phase C18 HPLC (LC-6AD, Shimadzu, Japan) assay after the pH was adjusted 7.4. 180 mM of KH_2_PO_4_ (5% methanol) was used as mobile phase (pH 6.25) running at 0.8 ml/min. The assay was linear from 0.05 to 200 μg/mL for ATP with coefficient of determination (R^2^) >0.999. Validation coefficients of variation for intra- and inter-day assays were less than 1.5% and 5.1%, respectively. Relative ATP content is calculated according to the peak area *versus* the ATP stand curve.

### Cell viability assay

The effects of drugs on the cell viability were determined by the MTS assay (CellTiter 96® AQueous One Solution Cell Proliferation assay, Promega Corporation, Madison, WI, USA) as reported previously [9]. Briefly, cells were cultured in 96-well plates and treated with indicated agents for 24 or 48 h. Then treated cells were incubated with 20 μL of MTS for additional 3 h. The absorbance was measured at 490 nm with Automatic microplate reader (Sunrise, Tecan). Cell viability was calculated by the following formula: cell viability (%)  =  (average absorbance of treated group – average absorbance of blank)/(average absorbance of untreated group- average absorbance of blank)]×100%.

### Histone and protein acetylation *in vitro* and in cultured cells

For *in vitro* protein acetylation, cell lysate from either cancer cells or mouse thymocytes were incubated with various doses of LC and TSA or Buty in a HDAC assay buffer at 37°C for 1.5 h, then acetylated-H3, -H4, and lysine-acetylated proteins were detected by Western Blot. For protein acetylation in cultured cells, either cancer cell or mouse thymocyte was treated with different concentrations of LC or TSA and Buty for various time points, cells were collected and then protein acetylation was detected by Western Blot.

### Western Blot

Western blot was performed as described previously [11], [12]. Briefly, an equal amount of total protein extracted from cultured cells was separated by 12% SDS-PAGE and transferred to polyvinylidene difluoride (PVDF) membranes. Primary antibodies and horseradish peroxidase (HRP)-conjugated appropriate secondary antibodies were used to detect the designated proteins. The bounded secondary antibodies on the PVDF membrane were reacted to the ECL detection reagents and exposed to X-ray films (Kodak, Japan). The x-ray film exposure was scanned and digitalized using a high-resolution scanner. The density of desired bands was quantified with the Quantity One software (BioRad).

### Cell cycle analysis

Cell cycle analysis was performed as reported previously [12]. HepG2 cells were seeded in 6-cm dishes overnight in RPMI 1640 medium supplemented with 10% fetal bovine serum, then treated with LC at indicated time points. The cells were collected, washed by PBS, and stained with PI in the presence of RNAase. Data were analyzed based on the distribution of cell populations in different phases of cell cycle.

### Computational modeling

In order to understand the molecular interaction between L-carnitine and HDAC, a molecular docking study was carried out with CDOCKER protocol of Discovery Studio 2.0 (Accelrys Software Inc). The crystal structure of HDAC (PDB ID: 1ZZ0) was used as the docking protein. First, only the chain A subunit was maintained after other chain subunits, alternate conformations, the original ligand ACT, Potassium metals and water molecules were removed. Then, hydrogen atoms and Gasteiger charges were added to the subunit. Finally, only hydrogen positions were optimized with Dreiding-like forcefield using the Clean Geometry menu. Meanwhile, the compound L-carnitine selected as the docking inhibitor was also optimized with Dreiding-like forcefield using the Clean Geometry menu. The protein HDAC was rigid, while the ligand LC was flexible during the docking process. The Input Site Sphere was centered on the original ligand ACT with radius 12 Å. Top hits, Random conformations and Orientations to refine parameters were all set to 20. The conformation corresponding to the lowest CDOCKER Interaction Energy was selected as the most probable binding conformation. At last, the docking complex was further estimated binding free energy by the Calculate Binding Energies protocol. All parameters used in calculation were default except for explained.

### HDAC activity assay *in vitro* and in cultured cells

HDAC activity *in vitro* and in cultured cells was detected with The HDAC-Glo™ I/II Assay and Screening System (Promega, USA) following the standard protocol. HDAC assay was performed in white 96-well plate. For *in vitro* HDAC activity assay, cell lysate (optimal protein content: 1 µg) was incubated with various agents in a HDAC assay buffer (100 µl) at 37°C for 60 mins and then 100 µl HDAC^TM^ I/II reagent was added, after 30 mins, luminescence was measured. For HDAC assay in cultured cells, cells (10000 cells/well) were treated with LC or positive control agents for 6 h or 12 h, then cell medium was changed to serum-free medium plus HDAC buffer (50 µl+50 µl). After 15 min incubation, HDAC^TM^ I/II reagents (100 µl) were added to the cells, luminescence was measured after 30 mins.

### Quantitative real-time PCR

Total RNAs were extracted from HepG2 cells with TRIzol reagent. Reverse transcription of purified RNA was performed using superscript III reverse transcription according to the manufacturer's instructions (Invitrogen). Quantification of all gene transcripts was done by quantitative using the TaKaRa SYBR Premix Ex Taq kit with Applied Biosystems 7500 Fast Real-Time PCR system. The values of P21 and P27 were shown against the value of GAPDH which was used as a control. The primer sets for amplification are listed below: p21-F: 5′GTC CAG CGA CCT TCC TCA TCCA3′; p21-R: 5′CCA TAG CCT CTA CTG CCA CCA TC3′; p27-F: 5′ACT GAG GCG GAG ACG AAG GT3′; p27-R: 5′CCT GAC AAG CCA CGC AGT AGAT3′; GAPDH-F: 5′CCA GCA AGA GCA CAA GAG GAA3′; GAPDH-R: 5′GGT CTA CAT GGC AAC TGT GAGG3′.

### ChIP assays

1×10^7^ HEPG2 cells were prepared for the ChIP assay, performed as described previously [13] by KangChen biotech company (Shanghai). For each ChIP assay, the sample was a mixture of 3 independent cell samples. Anti-H3K9 antibody was used to immunoprecipitate histones. All ChIP samples were done by real-time PCR, using the TaKaRa PCR Thermal Cycler and Rotor-Gene 3000 Realtime PCR. P21 and p27 primers were as follows: p21-F: 5′GCC GAA GTC AGT TCC TTG TG3′; p21-R: 5′CGG GGT CCC CTG TTG TCT3′; p27-F: 5′CTC TGA GGA CAC GCA TTT GGT3′; p27-R: 5′TGC AGG TCG CTT CCT TAT TC3′. Data are presented as fold enrichment calculated by each antibody ChIP value (IP/Input, the percentage of input) relative to IgG control ChIP value.

### DNA microarray assay and analysis

HepG2 cells were treated different doses of LC for 24 h, and then cells were collected and extracted with TRIzol agents. RNA quantity and quality were measured by NanoDrop ND-1000. RNA integrity was assessed by standard denaturing agarose gel electrophoresis. DNA microarray was performed by KangChen biotech company (Shanghai) in compliance to MIAME guidelines. The Human 12×135K Gene Expression Array was manufactured by Roche NimbleGen. Briefly, About 5 μg total RNA of each sample was used for labeling and array hybridization as the following steps: (1) Reverse transcription with by Invitrogen Superscript ds-cDNA synthesis kit; (2) ds-cDNA labeling with NimbleGen one-color DNA labeling kit; (3) Array hybridization using the NimbleGen Hybridization System and followed by washing with the NimbleGen wash buffer kit; (4) Array scanning using the Axon GenePix 4000B microarray scanner (Molecular Devices Corporation). Scanned images (TIFF format) were then imported into NimbleScan software (version 2.5) for grid alignment and expression data analysis. Fold increases of gene expression were calculated compared to the vehicle control.

### Statistical methods

Unless indicated otherwise, Mean+SD are presented where applicable. Unpaired Student's *t*-test or one way ANOVA is used where appropriate for determining statistic probabilities. *P* value less than 0.05 is considered significant.

## Results

### LC treatment selectively inhibits cancer cell growth *in vivo* and *in vitro*

**Figure 1:**
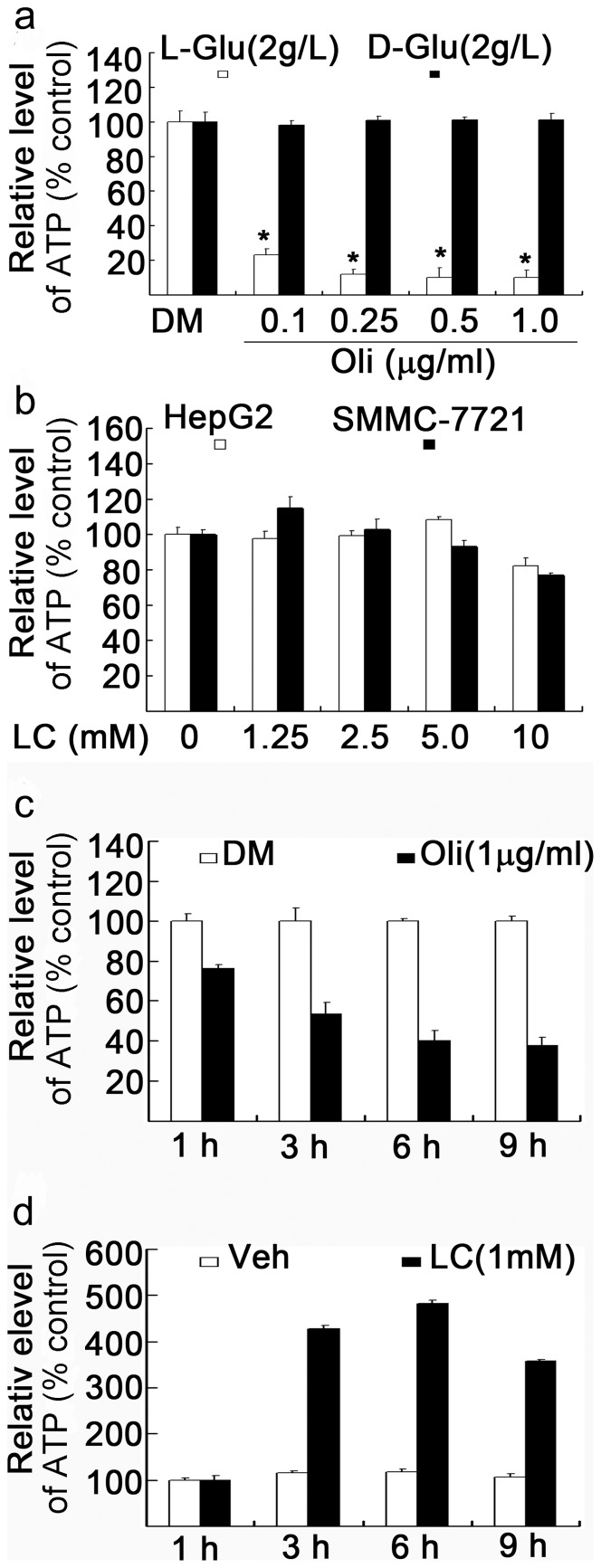
L-carnitine treatment fails to increase ATP concentration in cancer cells. (**a**) Cancer cells are resistant to oligomycin in the presence of D-glucose (2 g/L) but not L-glucose (2 g/L). Human HepG2 cancer cells were cultured in the presence or absence of either D-glucose or L-glucose in the culture medium and treated with various doses of oligomycin (0.1, 0.25, 0.5. 1.0 µg/ml) for 6 h and ATP content was assessed. Mean+SD (n = 3). *P<0.01, *versus* control. DM: DMSO. (**b**) LC does not increase intracellular ATP content in cancer cells. Human hepatic HepG2 and SMMC-7721 cells were cultured in the normal culture medium respectively and treated with different doses of LC for 6 h, ATP content was detected. LC: L-carnitine. (**c**) Thymotytes are sensitive to oligomycin in the presence of D-glucose (2 g/L). Mouse thymocytes were treated with oligomycin (1 mg/ml) for different time points (1, 3, 6, 9 h), total ATP content was detected. (**d**) LC efficiently increases cellular ATP content. Mouse thymocytes were treated with LC (1 mM) for various times, cellular ATP content was assassed. Veh: vehicle.

It has been well known that most cancer cells depend on glycolysis for ATP generation. To further confirm that in our cell systems, multiple cancer cell lines were used to test whether oligomycin could inhibit ATP production. The result in representative cancer cells was shown. It was found that in the presence of L-glucose in the culture medium, oligomycin quickly depleted ATP production while in the presence of D-glucose in the culture medium oligomycin did not affect ATP generation (Fig. 1*a*). Since L-glucose can not be used to produce ATP production [10], this result implies that cancer cells mainly rely on D-glucose glycolysis for ATP production, consistent to previous reports [4], [5]. We next investigated whether LC treatment could increase intracellular ATP production in cancer cells. Consistent to our prediction, LC treatment could not further increase ATP production in both hepatic HepG2 and SMMC-7721 cancer cells (Fig. 1*b*). However, in contrast to the effect in cancer cells, oligomycin, when added in mouse thymocytes cultured with D-glucose, time-dependently inhibited ATP generation (Fig. 1*c*) while LC efficiently increased intracellular ATP content at different time points under this condition (Fig. 1*d*). These results confirm that LC is able to generate ATP in normal mouse thymocytes, but not in hepatic HepG2 and SMMC-7721 cancer cells.

**Figure 2:**
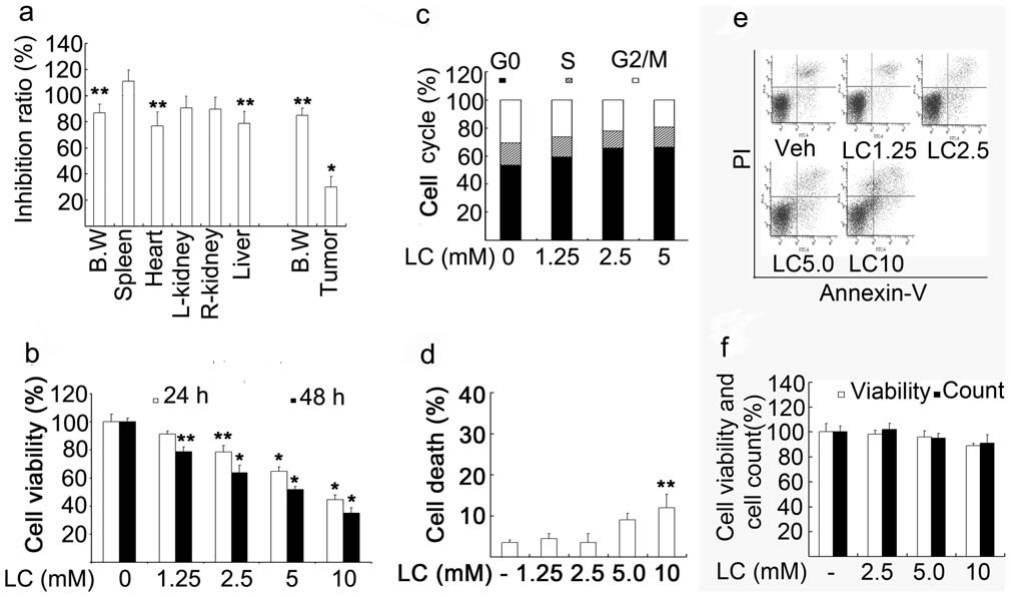
LC treatment inhibits cancer cell proliferation *in vivo* and *in vitro*. (**a**) LC selectively inhibits HepG2 tumor growth compared with normal tissues. BALB/c nude mice were *s.c*. inoculated in the left armpit of each mouse with HepG2 cells (1×10^6^ cells/mouse). When the tumor size reaches 50–75 mm^3^, nude mice were *i.p*. injected with 400 mg/kg for consecutive 15 days. Normal Balb/c mice were treated as the nude mice. Body and organ weight were detected. B. W: Body weight. Mean+SD (n = 8). *P<0.01, **P<0.05, *versus* each control respectively. % inhibition = body weight or organ weight in the LC-treated group/ average body weight or organ weight in the control group×100. (**b**) LC inhibits HepG2 cell proliferation in a dose-dependent manner. HepG2 cells were treated with various doses of LC (1.25, 2.5, 5, 10 mM) for 24 h or 48 h, cell proliferation was detected by MTS assay. Mean+SD (n = 3). *P<0.01, **P<0.05, compared with the control. (**c**) LC induces cell cycle arrest at Go/G1 phase. HepG2 cells were treated with different doses of LC for 24 h, cell cycle were detected by flow cytometry. Representative results were shown. (**d, e**) LC slightly induces cell death. HepG2 cells were exposed to various doses of LC for 24 h and cell apoptosis was detected by flow cytometry. Summary of the data were shown in (**d**) and representive flow images were shown in (**e**). **P<0.05, versus control. (**f**) LC does not dramatically affect thymocyte cell viability. Mouse thymocytes were treated with various doses of LC (2.5, 5, 10 mM) for 24 h, cell viability was detected by MTS assay and cell number was count.

Next we compared the effects of LC on normal tissue and cancer growth *in vivo*. Normal Balb/c mice or Balb/c nude mice inoculated with HepG2 cancer cells were *i.p*. injected with LC (400 mg/kg) for 15 days except day 8, followed by termination of the experiment. This dose schedule is a tolerated dose for Balb/c nude mice. Organ weight and tumor weight of each mouse treated by LC or control were compared. It was found that LC treatment inhibited more than 70% of cancer growth, while the same treatment decreased less than 20% of the normal organ development and body weight (Fig. 2*a*).

We then investigated the effects of LC on cell proliferation by MTS assay. It was found that LC dose-dependently decreased HepG2 cell viability (Fig. 2*b*), associated with the cell cycle arrest at the G0/G1 phase (Fig. 2*c*) and low levels of cell death (Fig. 2, *d* and *e*). These results implied that LC predominantly induced cell proliferation inhibition but slightly induced cell death in cancer cells. Finally, in normal mouse thymocytes, LC had little effect on thymocyte viability and cell count within 24 h treatment (Fig. 2*f*). These results confirm that LC selectively induced cancer cell cytotoxicity in *vitro* and *in vivo*.

### LC treatment induces the expression of p21^cip1^ gene, mRNA and protein in cancer cells

**Figure 3:**
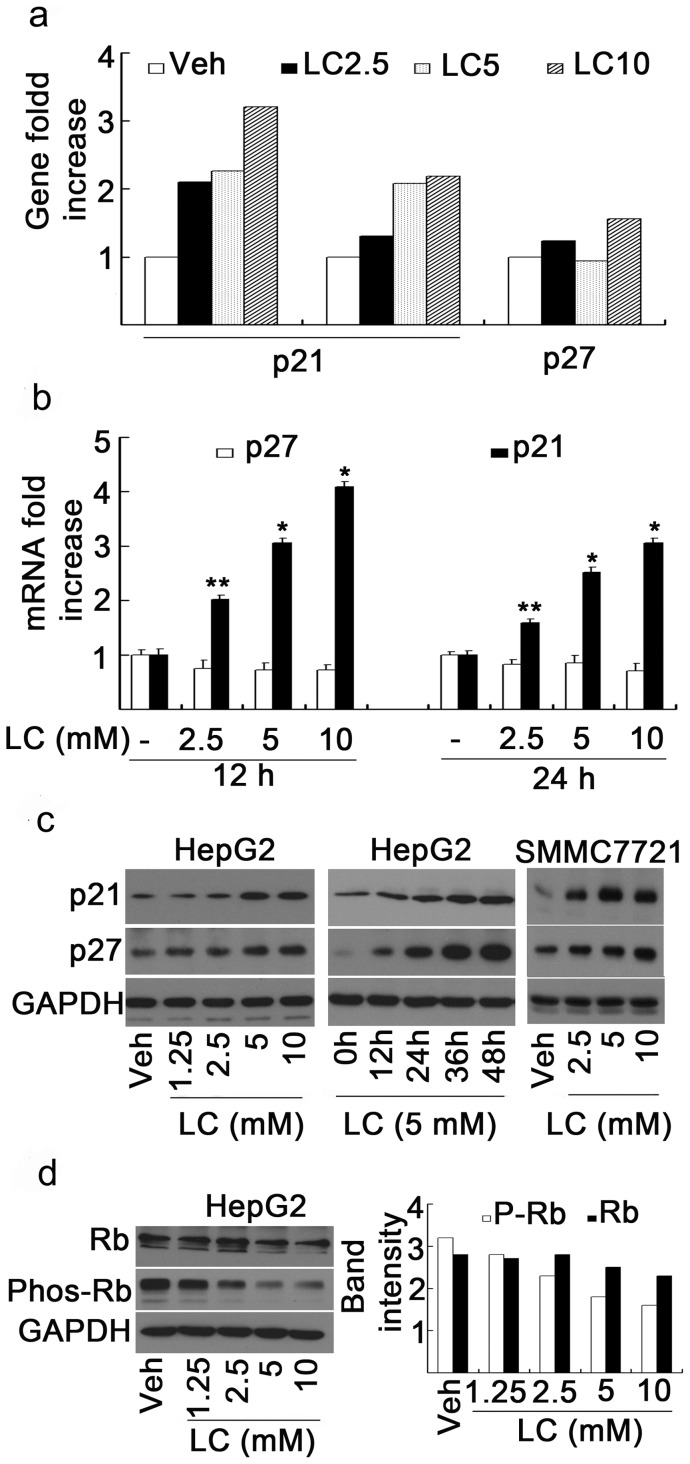
LC treatment selectively induces expression of p21^cip1^ gene, mRNA and protein in cancer cells. (**a**) LC induces p21^cip1^ gene expression but not p27 and GAPDH. HepG2 cells were treated with LC (2.5, 5.0, 10 mM) for 24 h; cells were collected for gene expression profile analysis. In the gene chip, there are 2 probes for p21^cip1^ and 1 probe for p27^kip1^. All the fold increases of p21^cip1^ and p27^kip1^ gene expression *versus* control were shown. (**b**) LC dose-dependently induces p21^cip1^ mRNA expression but not p27^kip1^ in HepG2 cells. HepG2 cells were incubated with different concentrations of LC (2.5, 5, 10 mM) for either 12 h or 24 h; the cells were collected for mRNA assay of p21^cip1^ and p27^kip1^ by real-time PCR. Fold increase of the LC-treated *versus* control was shown. Mean+SD (n = 3). *P<0.01, **P<0.05, compared with control. (**c**) LC dose-dependently and time-dependently induces p21^cip1^ protein accumulation in HepG2 cancer cells. HepG2 and SMMC7721 cells were treated with various doses of LC for 48 h or HepG2 cells were exposed to 5 mM of LC for 12, 24, 36, 48 h; p21 and p27 proteins were detected by Western blot. (**d**) LC dose-dependently decreases Rb phosphorylation. HepG2 cells were treated with LC for 48 h; Rb and phosphorylated Rb were dectected by Western blot. Typical Western images were shown (left) and band intensity was quantified (right).

We next determined the molecular mechanism for how LC could selectively inhibit tumor growth. To do so, the gene expression profile was investigated in HepG2 cells after three doses of LC treatment (2.5, 5, and 10 mM) for 24 h. All the up-regulated and down-regulated genes (at least 2 fold increase or decrease at all the three doses) after LC treatment were summarized (data not shown). Among the up-regulated genes, p21^cip1^ gene but not p27^kip1^ gene, two important genes associated with cell cycle regulation, was found to be highly and dose-dependently expressed (Fig. 3*a*). The effects of LC treatment on p21^cip1^ and p27^kip1^ mRNA levels were next determined by real-time PCR. HepG2 cells were cultured with or without LC (2.5, 5, 10 mM) for either 12 h or 24 h, RNA were prepared from the cells. After treatment with LC, the p21^cip1^ mRNA level dose-dependently increased both at 12 h and 24 h time points, and p21^cip1^ mRNA level is relatively higher after 12 h treatment than 24 h treatment, while p27^kip1^ mRNA did not show much change at these two time points (Fig. 3*b*). To determine the protein levels of p21^cip1^ and p27^kip1^, Western blot assay was performed in the treated HepG2 cells. The results showed that after treatment with various doses of LC for 48 h, p21^kip1^ protein levels increased in a dose-dependent manner in HepG2 cancer cell lines (Fig. 3*c*, *left*). Further dynamic study in HepG2 cells showed that after treatment with LC (5 mM) for 12, 24, 36, and 48 h, p21^cip1^ time-dependently increased (Fig. 3*c*, *middle*). In SMMC7721 cells LC treatment also increased p21 protein expression in a dose-dependent manner similar to in HepG2 cells (Fig. 3*c*, *right*). Unexpectedly, p27 protein increased in both dose- and time-dependent manners after LC treatment (Fig. 3*c*) even though p27^kip1^ mRNA level is stable, suggesting that LC might inhibit the degradation of p27 protein. To further investigate the effect of LC on the downstream effects of p21^cip1^ and p27^kip1^, levels of unphosphorylated Rb and phosphorylated Rb (phos-Rb) were detected. It was found that LC treatment slightly decreased Rb protein but dramatically decreased Phos-Rb protein (Fig. 3*d*). These results demonstrated that LC selectively induced p21^cip1^ expression.

### LC treatment increased histone acetylation in cultured cells

**Figure 4:**
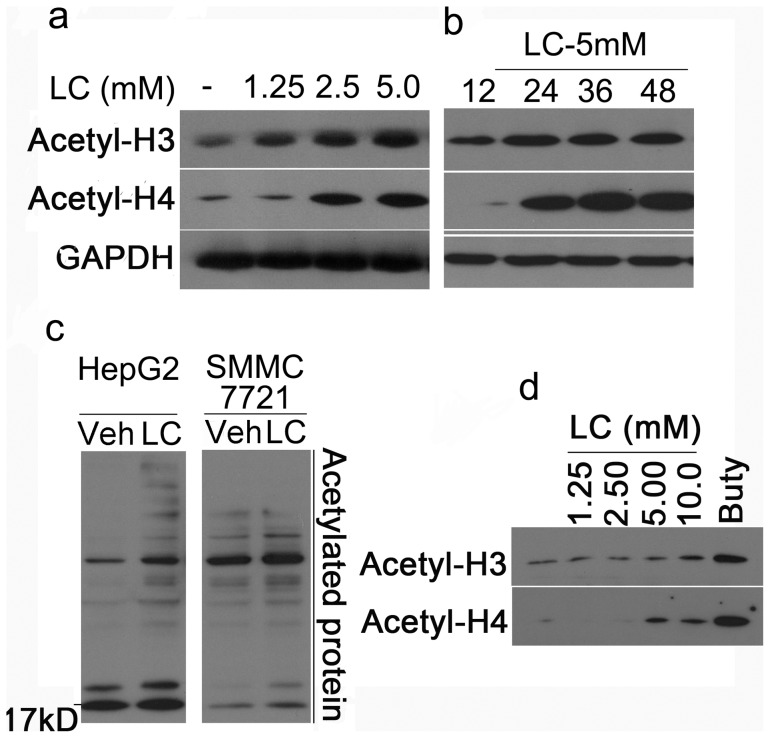
LC induces Histone acetylation in cultured cells. (**a**) LC dose-dependently induces accumulation of acetylated histones. HepG2 cells were exposed to LC (1.25, 2.5, 5.0 mM) for 48 h, acetylated histones H3 and H4 were detected by Western blot. GAPDH was used as a loading control. (**b**) LC time-dependently induces accumulation of acetylated histones. HepG2 cells were treated with LC (5 mM) for different time points (12 h, 24 h, 36 h, 48 h) and acetylated histones were detected. (**c**) LC treatment increases lysine-acetylated protein accumulation in human HepG2 and SMMC-7721 cancer cells. Human HepG2 and SMMC-7721 cells were treated with LC (10 mM) for 12 h, and then cells were collected for Western blot to detect acetylated proteins with lysine acetylated antibody. (**d**) LC dose-depentently increases accumulation of acetylated histones in mouse thymocytes. Mouse thymocytes were treated with LC for 24 h, histone acetylation was detected by Western Blot. Buty (1 mM) was used as a positive control.

Based on Warburg's theory, we hypothesized that supplement of LC in cancer cells would disturb protein acetylation. Previous reports have shown that histone acetylation by HDAC inhibitors selectively induced p21^cip1^ expression but not p27^kip1^ [14], [15]. Our results have demonstrated that LC selectively induced p21^cip1^ expression but not p27^kip1^ (Fig. 2). Therefore, we investigated the effects of LC on histone acetylation and accumulation of lysine-acetylated proteins in cultured cells. HepG2, SMMC-7721 cancer cells and mouse thymocytes were treated with LC. As shown in Fig. 4, *a* and *b*, LC increased acetylation of histone H3 and H4 in a dose- and time-dependent manner in HepG2 cancer cells. LC could also induce accumulation of lysine-acetylated proteins in both HepG2 and SMMC-7721 cancer cells (Fig. 4*c*). In primary thymocyte culture, LC dose-dependently increased histone acetylation similar to in cancer cells (Fig. 4*d*). These results suggest that LC treatment increased protein (histone) acetylation in cultured cells.

### LC directly inhibits HDAC activities *in vitro* and in cultured cells

**Figure 5:**
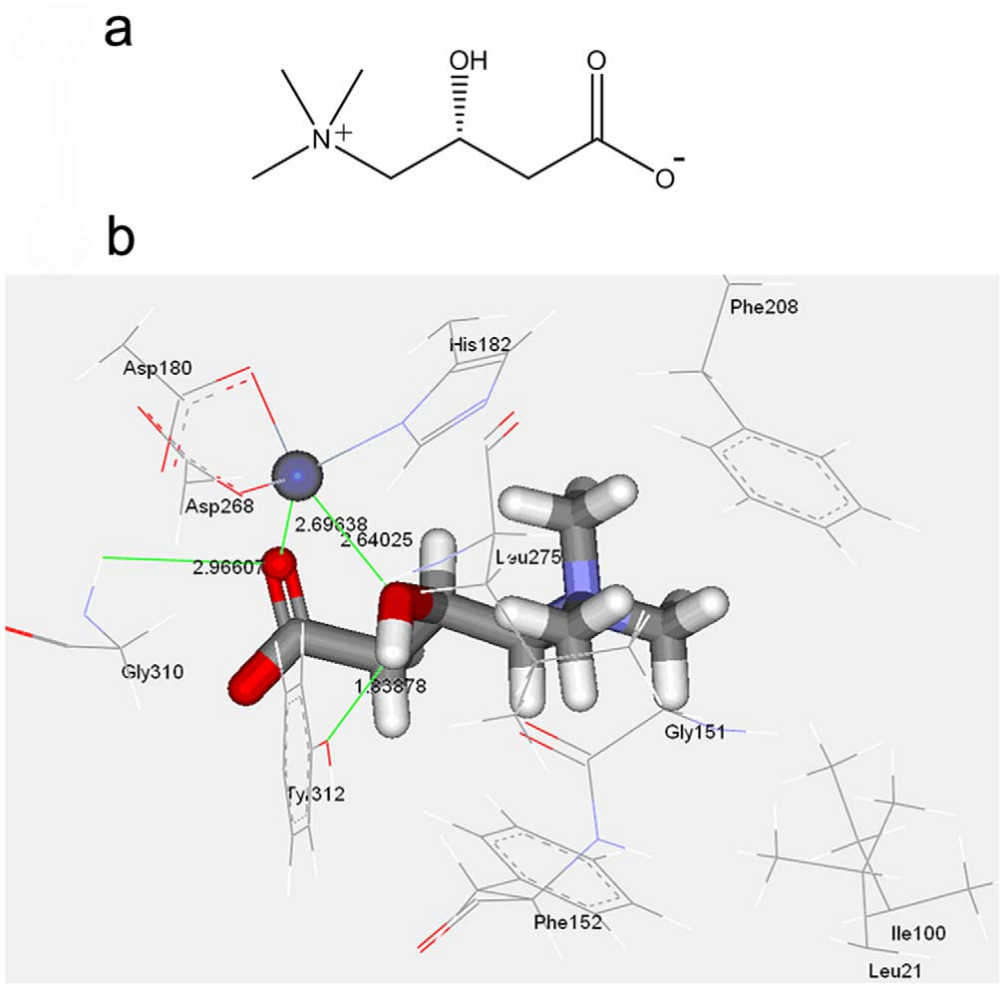
Computional molecular docking of LC with HDAC. (**a**) The chemical structure of LC was shown. (**b**) The docking model of L-carnitine in active site of HDAC.

Since LC induces histone acetylation, we then investigated whether LC could directly affect HDAC activities. A computational docking model was first established. The crystal structure of an HDAC-like protein from the hyperthermophilic bacterium *Aquifex aeolicus* with the HDAC inhibitor TSA has been reported [16]. The structure shows the position of the essential zinc atom that is involved in catalysis of class I/II HDAC. The chemical structure of LC and the docking model of complex (L-carnitine and HDAC) were shown in Fig. 5, *a* and *b*, and its CDOCKER Interaction Energy and Binding Energy were −29.01 and −85.79 kcal/mol, respectively. Fig. 5*b* shows that one oxygen atom of carboxyl anion and oxygen atom of hydroxyl form coordination interactions with Zinc ion, with distances of 2.696 Å and 2.640 Å, respectively. This interaction model is different in comparison with original ligand ACT with its two oxygen atoms of carboxyl anion coordinated to Zinc ion. The carboxyl anion formed a weak hydrogen bond with Gly310, with distance of 2.966 Å, and the hydroxyl formed a hydrogen bond with Tyr312, with distance of 1.839 Å. In addition, the (CH_3_)_3_N^+^ group located in a hydrophobic entryway and interacted with the hydrophobic surface composed of the side chains of Leu21, Ile100, Phe152, Phe208 and Leu275. Moreover, the group formed cation-π interactions with the benzene rings of Phe152 and Phe208. This model predicted that LC has the potential to interact with HDAC active sites.

**Figure 6:**
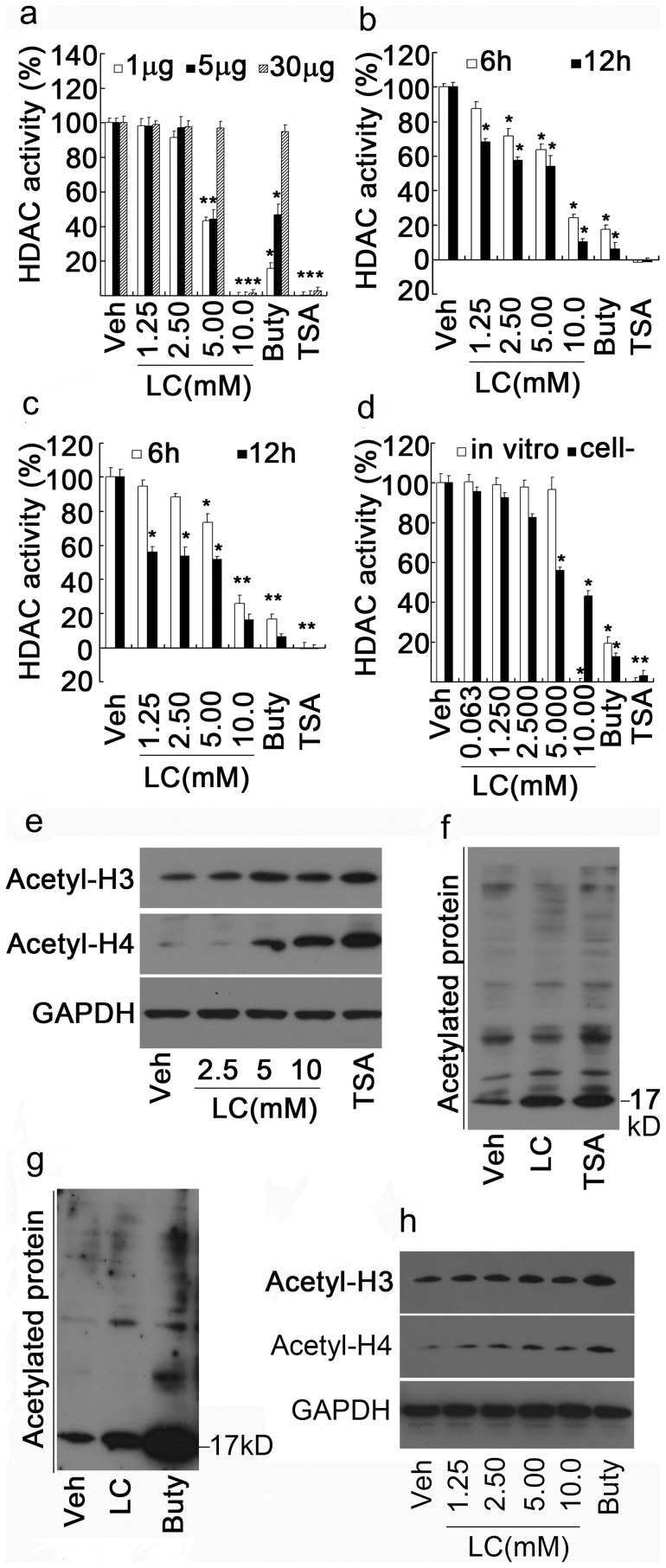
LC directly inhibits HDAC activity *in vitro* and in cultured cells. (**a**) LC directly inhibits HDAC activity *in vitro*. HepG2 cell lysates (1, 5, 30 µg protein) were treated with various doses of LC for 1 h, HDACI/II activity was detected. HDAC inhibitors Buty and TSA were used as positive controls. *P<0.05, versus vehicle control. (**b, c**) LC inhibits HDAC activites in cultured cells. HepG2 and SMMC-7721 cancer cells were treated with various doses of LC for either 6 h or 12 h, HDACI/II activities were detected. Buty and TSA were used as positive controls. *P<0.05, versus vehcile control. (**d**) Acetyl-LC inhibits HDAC1/2 activities *in vitro* and in cultured cells. HepG2 cells and cell lysates were exposed to various doses of Acetyl-LC for 6 h and 1 h respectively, HDACI/II activities were detected. Cell-: in cultured cells. (**e, f**) LC directly increases histone acetylation and lysine-acetylated protein accumulation *in vitro*. HepG2 cell lysates were treated with LC for 1.5 h, Acetyl-H3, –H4 and lysine-acetylated proteins were detected by Western Blot. TSA (1 µM) was used as o pisitive control. (**g, h**) As in (**e, f**), mouse thymocyte lysates were used instead of HepG2 cell lysates. Buty (1 mM) was used as a control.

To confirm this computer prediction, the direct effect of LC on activities of classical I and II HDAC were examined *in vitro*. Cell lysates (1, 5, 30 µg) from HepG2 cells were treated with various doses of LC and TSA or Buty. As shown in Fig. 6*a*, TSA (1 µM), the strongest HDAC inhibitor, completely inhibited HDAC activities, and Buty, the relatively weak HDAC inhibitor, dramatically inhibited HDAC activity at low HDAC concentration (1 µg protein) but with the increase of HDAC donor (5, 30 µg protein), the inhibiting effect of Buty (1 mM) on HDAC activity gradually declined. Similar to Buty, 5 mM dose of LC could efficiently inhibited HDAC I/II activities while 10 mM LC completely inhibited HDAC activities.

To confirm this effect in cultured cells, HepG2 and SMMC-7721 cancer cells were treated with various doses of LC and the positive controls for 6 h or 12 h, HDAC I/II activities were detected. Similar to the *in vitro* results, LC dose-dependently inhibited HDAC activities in these cultured cancer cells as well (Fig. 6, *b* and *c*). To further verify the effects of LC, a modified form of LC, acetyl-L-carnitine (Acetyl-LC), was used to detect its effect on HDAC activity. As shown in Fig. 6*d*, acetyl-LC, similar to LC, could also inhibit HDAC activities both *in vitro* and in cultured cells. 10 mM dose of Acetyl-LC completely inactivated HDAC I/II activity *in vitro*.

### LC increases histone acetylation *in vitro*

Since LC could directly inhibit HDAC activities, we next investigated the effects of LC on protein acetylation *in vitro*. To do so, cell lysates from either HepG2 cells or mouse thymocytes were treated with various doses of LC and positive control agents. As shown in HepG2 cell lysate, LC dose-dependently increased acetylation of H3 and H4 (Fig. 6*e*), and similar to TSA, LC (10 mM) also induced accumulation of lysine-acetylated proteins (Fig. 6*f*); By using mouse thymocyte lysate, LC had the similar effects on histone acetylation and lysine-acetylated proteins (Fig. 6, *g* and *h*). These results demonstrated that LC could directly increase histone acetylation *in vitro via* inhibiting HDAC activities.

LC treatment induces accumulation of acetylated histones in chromatin associated with the p21^cip1^ but not p27^kip1^ promoter gene.

**Figure 7:**
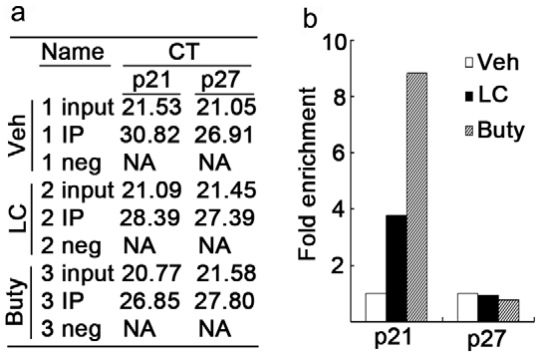
LC treatment induces accumulation of acetylated histones in chromatin associated with p21^cip1^ gene but not p27^kip1^ gene. (**a, b**) HepG2 cells were treated with LC (10 mM) and Buty (1 mM) for 12 h; cells were collected for CHIP assay as described in the Materials and Methods part. The PCR data and fold enrichment of p21^cip1^ and p27^kip1^ promoter gene in LC- or Buty-treated *versus* vehicle control were shown in (**a**) and (**b**) respectively. IP: immunoprecipitation.; Neg: negative.

To study whether LC-induced p21^cip1^ expression is associated with histone acetylation, the effect of LC on the acetylation of histone H3 associated with the p21^cip1^ promoter gene was then examined by using ChIP. Chromatin fragments from HepG2 cells cultured with or without LC (10 mM) for 12 h were immunoprecipitated with an antibody to acetylated histone H3K9. DNA from the immunoprecipitate was isolated. Approximately 4-fold enrichment of p21^cip1^promoter gene, but not p27^kip1^ was associated with acetylated histone in the cells treated with LC, compared with the same region isolated from cells cultured without LC (Fig. 7, *a* and *b*). Buty (1 mM) induced more than 8-fold enrichment of p21^cip1^ promoter gene but not p27^kip1^ (Fig. 7, *a* and *b*).

## Discussion

Many mechanisms have been reported to be involved in HDAC inhibition-induced cytotoxicity [17], [18]. HDAC inhibitors not only inhibit cell proliferation but also induce cell death once the inhibition is strong enough.

It is well known that p21^cip1^ is a cyclin-dependent kinase inhibitor that directly inhibits the activity of cyclin E/CDK2 and cyclin D/CDK4/6 complexes, thus inhibiting Rb phosphorylation. p21^cip1^ functions as a regulator of cell cycle progression at S phase. Highly expressed p21^cip1^ would inhibit cell proliferation. What is interesting is that even though the expression of p27^kip1^ gene and mRNA remained unchanged after LC treatment, p27^kip1^ protein level dose- and time-dependently increased with LC treatment. p27^kip1^ protein, like p21^cip1^, increased at a relatively low dose and at an early time point, which implied that p27 protein accumulation is possibly regulated at a post-translational level. Previous studies have reported that protein modification by phosphorylation or acetylation would affect the stability of modified proteins [19], [20]. It is a general mechanism by which protein acetylation or sumoylation modulates ubiquitination-dependent proteasome proteolysis [21], [22]. For example, both acetylation and ubiquitination can modify the same lysine residues at the C terminus of p53, implicating a role of acetylation in the regulation of p53 stability [23]. Since LC could induce the acetylation of multiple proteins, protein acetylation by interfering protein ubiquitination could be one of the mechanisms to affect p27 protein degradation, thus inducing p27 accumulation, which need to be confirmed in the future study. As shown, LC could dose-dependently decreased Rb phosphorylation, a downstream target of both p21 and p27 protein. Therefore, p27 protein accumulation together with p21^cip1^ high expression would contribute to LC-mediated cytotoxicity.

However, we have also noticed that after LC treatment increased not only histone protein acetylation but also other protein acetylation as well (Fig. 4*c*), which suggests that other mechanisms are possibly involved in LC-mediated cytotoxicity in cancer cells. In recent years, protein acetylation has emerged as a major posttranslational modification for proteins [24], [25]. The regulatory scope of lysine acetylation is broad and comparable with that of other major posttranslational modifications including protein phosphorylation, sumoylation, ubiquitination and methylation [25]. Lysine acetylation is a reversible posttranslational modification of proteins and plays a key role in regulating gene expression. 3600 lysine acetylation sites on 1750 proteins and quantified acetylation changes in response to the deacetylase inhibitors have been identified [24]. It is found that lysine acetylation preferentially targets large macromolecular complexes involved in diverse cellular processes, such as chromatin remodeling, cell cycle, nuclear transport, splicing, and actin nucleation [24]. Other study further confirmed that virtually every enzyme in glycolysis, gluconeogenesis, the tricarboxylic acid cycle, the urea cycle, fatty acid metabolism, and glycogen metabolism was found to be acetylated in human liver tissue, suggesting that lysine acetylation plays a major role in cell metabolisim and cell viability [26].

We have confirmed that LC is a HDAC inhibitor. It was found that LC treatment mainly arrested cancer cell proliferation and only slightly induced cell death, which is possibly due to the following two reasons: on one hand, LC is not a strong HDAC inhibitor compared to classical inhibitors including TSA but LC and Buty exerted their inhibiting effects at a similar mM level; on the other hand, relative low level of LC could efficiently induced high expression of p21^cip1^ which has been reported to block HDAC inhibition-induced apoptosis [27], [28].

We found that normal thymocytes are more resistant to LC treatment-induced cytotoxicity than cancer cells (Fig. 2). In cancer cells, LC treatment dose-dependently decreased cell viability but in normal thymocytes, LC exerted very weak effect on cell viability. These results are consistent to previous reports by using HDAC inhibitor TSA [29]. TSA more selectively induced cancer cytotoxicity than normal cells. This difference has been verified in the *in vivo* experiment (Fig. 2*a*). Both *in vitro* and in cultured cells, it was found that LC treatment not only inhibited HDAC activities and induced histone acetylation in cancer cells but also in normal cells, but the cytotoxicity induced in normal and cancer cells are different. Therefore, even though the mechanism is unclear, the active states of HDAC in the cells are possibly responsible for the difference [29]. As regard to the different effects of LC on normal and tumor tissues, besides the sensitivity to HDAC inhibition, other mechanisms are possibly involved in this difference. Since normal lymphocytes were sensitive while cancer cells were resistant to oligomycin treatment for ATP generation, this implies that in cancer cells, the oxidative phosphorylation system worked in normal cells but not in cancer cells consistent to previous reports [30], [31]. In normal thymocytes, LC treatment efficiently induced ATP generation, indicating that LC could be used in normal cells, while in cancer cells LC treatment failed to generate ATP, indicating that LC could not be used for ATP generation. Therefore, reasonably LC would have more potential to affect other targets like HDAC in cancer cells than in normal cells. Even though in cultured cancer cells LC could slightly induced cell death, no cell death in tumor tissues was found like in other normal tissues (data not shown), this is possibly due to that the LC concentration *in vivo* is not as high as *in vitro*.

LC has been recognized to play an important role in cellular energy metabolism. The current study has found that HDAC is a new molecular target of LC.

Many studies have been done to determine the effectiveness of LC for fat burning. Also it has been clinically used in cancer patients with fatigue and carnitine deficiency [32], [33]. It has been reported that a deficiency of LC is a risk factor for liver cancer. Furthermore, it was found that long-term LC supplementation may prevent the development of liver cancer [34], [35]. Even though it has been used under many clinical conditions, the mechanism is still unclear. HDAC inhibitor has been developed as anti-cancer drugs [36], [37]. It has been reported previously that histone acetylation mediated by HDAC inhibition could block cell proliferation and induce cell death [17], [18], [38]. These data verified that LC mediated histone acetylation *via* inhibiting HDAC which at least partially contributed to its cytotoxicity. Therefore, LC would be promising in cancer therapeutics.

LC is not as strong as other HDAC inhibitors like TSA, therefore, LC alone may not be a more potent anti-cancer agent than other HDAC inhibitors, but the importance relies on that LC is an intracellular molecule well known to transport acyl CoA for ATP production under physiological conditions. It has been reported that intracellular LC concentration is at a low mM level [39], [40], and this dose of LC is at a level to at least partially inhibit HDAC activities in most of the cells. We have predicted that LC has the potential to interact with HDAC, therefore, it is possible that the cellular LC and HDAC are in a binding state under physiological conditions, which is worth to be further investigated in the future work.
